# Diagnosis of giant cell arteritis by temporal artery biopsy is associated with biopsy length

**DOI:** 10.3389/fmed.2022.1055178

**Published:** 2022-11-28

**Authors:** Carlee Ruediger, Jem Ninan, Kathryn Dyer, Suellen Lyne, Joanna Tieu, Rachel J. Black, Thomas Dodd, Susan Lester, Catherine L. Hill

**Affiliations:** ^1^Rheumatology Unit, The Queen Elizabeth Hospital, Woodville South, SA, Australia; ^2^Discipline of Medicine, The University of Adelaide, Adelaide, SA, Australia; ^3^Rheumatology Unit, Northern Adelaide Local Health Network, Adelaide, SA, Australia; ^4^Rheumatology Unit, Royal Adelaide Hospital, Adelaide, SA, Australia; ^5^SA Pathology, Adelaide, SA, Australia; ^6^Basil Hetzel Institute, Woodville South, SA, Australia

**Keywords:** giant cell arteritis (GCA), biopsy, vasculitis, diagnosis, temporal arteritis

## Abstract

**Aims:**

Temporal artery biopsy (TAB) is a widely used method for establishing a diagnosis of Giant Cell Arteritis (GCA). The optimal TAB length for accurate histological GCA diagnosis has been suggested as 15 mm post-fixation (15–20 mm pre-fixation). The aim of this study was to determine the relationship between a histological GCA diagnosis and optimal TAB length in the South Australian (SA) population.

**Materials and methods:**

Pre-fixation TAB length (mm) was reported in 825/859 of all samples submitted to SA Pathology between 2014 and 2020 from people aged 50 and over. When more than one biopsy was taken, the longest length was recorded. Analyses of both TAB length and TAB positive proportions were performed by multivariable linear and logistic regression analysis, including covariates sex, age, and calendar year.

**Results:**

The median age of participants was 72 (IQR 65, 79) years, 549 (66%) were female. The TAB positive proportion was 172/825 (21%) with a median biopsy length of 14 mm (IQR 9, 18). Biopsy length (mm) was shorter in females (*p* = 0.001), increased with age (*p* = 0.006), and a small positive linear trend with calendar year was observed (*p* = 0.015). The TAB positive proportion was related to older age (slope/decade: 6, 95% CI 3.6, 8.3, *p* < 0.001) and to TAB length (slope/mm 0.6, 95% CI 0.2, 0.9, *p* = 0.002), but not sex or calendar year. Comparison of models with TAB length cut-points at 5, 10, 15, 20 mm in terms of diagnostic yield, receiver operating characteristics and Akaike Information Criteria confirmed ≥ 15 mm as an appropriate, recommended TAB length. However, only 383 (46%) of the biopsies in our study met this criteria. The diagnostic yield at this cut-point was estimated as 25% which equates to an expected additional 30 histologically diagnosed GCA patients.

**Conclusion:**

This study confirms that TAB biopsy length is a determinant of a histological diagnosis of temporal arteritis, and confirms that a TAB length ≥ 15 mm is optimal. Approximately half the biopsies in this study were shorter than this optimal length, which has likely led to under-diagnosis of biopsy-proven GCA in SA. Further work is needed to ensure appropriate TAB biopsy length.

## Background

Giant Cell Arteritis (GCA) is an autoimmune condition causing inflammation of medium and large blood vessels, known as vasculitis. GCA is the most common vasculitis affecting the elderly. When presented with a case suggestive of the diagnosis of GCA, there is a need to initiate further investigations to exclude or confirm the diagnosis, historically based on the criteria set out by the American College of Rheumatology (ACR) (1), and more recently, the additional use of ultrasound and other imaging modalities of affected blood vessels (2).

With a specificity of 100%, a temporal artery biopsy (TAB) with histopathology demonstrating necrotizing arteritis characterized by a predominance of mononuclear cell infiltrates or a granulomatous process with multinucleated giant cells, has been a mainstay for a diagnosis of GCA (3). The average diagnostic yield of TAB for GCA is estimated as 25% (IQR 17, 34) (4), with a recent meta-analysis estimating an average sensitivity of only 77% (5), although this was highly variable with estimates from individual studies ranging between 50 and 95%. Therefore, a negative TAB does not exclude disease. This may in part be attributable to the recognition of extra-cranial disease (large vessel vasculitis) as part of the GCA disease spectrum, and it has been suggested that TAB may have an even lower sensitivity in these patients (6). However, technical aspects of the TAB sampling may also contribute to a decreased sensitivity of TAB for GCA. One such aspect is the presence of “skip lesions,” where areas of normal pathology may be interspersed within inflamed sections of the artery, resulting in a false negative result. Indeed, retrospective and prospective examination of TAB specimens have identified skip lesions in TAB from 28% of people with temporal arteritis, with inflammatory foci as small as 330 microns identified (7). Because of these skip lesions, the length of the TAB segment is therefore important. Both the British Society for Rheumatology (8) and the European League Against Rheumatism (9) recommend a TAB length of at least 1 cm (10 mm) which is supported by multiple studies (10–12). Other studies have suggested a minimum TAB length of 5 mm (13, 14) or even 20 mm (15–17) is appropriate. Two studies, which more formally evaluated TAB length in relation to diagnostic sensitivity demonstrated that 1.5 cm (15 mm) was the change point for GCA diagnostic sensitivity (18, 19), concluding that the optimal TAB length for accurate GCA diagnosis is at least 15 mm post-fixation (15–20 mm pre-fixation), with greater lengths unlikely to provide significant additional diagnostic yield. In contrast, other studies have reported no relationship between TAB length and diagnostic yield (20–23).

The aim of this study was to determine the relationship between optimal TAB length and a histopathological diagnosis of GCA in the South Australian (SA) population.

## Materials and methods

We retrospectively analyzed the results of all TAB reports from January 2014 to December 2020 for biopsies from people aged 50 years and over submitted to the SA public health sector pathology laboratory (SA Pathology). A total of 859 biopsy reports were reviewed, with 825 (96%) reporting pre-fixation TAB length (mm). When more than one biopsy was taken, the longest length was recorded. Age at biopsy and sex information was also collected on all biopsies. The details of the biopsy report including presence of inflammatory cell infiltrate, presence of Giant cells, disruption of internal elastic media, intimal hyperplasia and involvement of vasa vasorum were taken into account. The final opinion of the pathologist determined the assignment of the biopsy into either positive or negative categories. Although the TAB reports were unstructured, a review of specific details reported in 90 positive TAB indicated that giant cells (*n* = 70, 78%), intimal hyperplasia (*n* = 68, 76%), and adventitial inflammation (*n* = 68, 76%) were frequent findings. Over 80% of positive TAB reported inflammatory infiltrates involving all layers of the temporal artery. Those reporting inflammatory infiltrates without the specific term “transmural infiltration” had other strong characteristics of GCA such as the presence of Giant Cells. A specimen with significant eosinophilic infiltrate was excluded.

Statistical analysis was performed using Statav16 (StataCorp LLC, TX, USA). Multivariable regression analyses were used to determine covariates for TAB length (linear regression), TAB length ≥ 15 mm (logistic regression), and TAB positivity (logistic regression). All analyses included additional covariates sex, age, and calendar year. Both linear and quadratic terms were evaluated for the linear covariates age and TAB length, but a quadratic term was only retained for age in the logistic regression model for a TAB positive outcome based on the Akaike Information Criterion (AIC). Results were interpreted as marginal population-averaged predictions of the outcome for each covariate, which for the logistic regression model for TAB outcome, was the predicted proportion of positive TAB results. Additional models for the TAB outcome were estimated with the TAB length covariate dichotomized at 5, 10, 15, and 20 mm, and these models were compared using the AIC, the area under the receiver operating curve (AUC-ROC), and diagnostic yield (positive predictive value, PPV).

## Results

The median age at biopsy of the 825 patients included in the study was 76 years, 549 (66%) were female, with 172 (21%) TAB positive (Table 1). The age and sex distribution of TAB positive patients were comparable to that of previous studies of biopsy-proven GCA in South Australia (24). Notably, the number of TAB performed in 2020 (*n* = 143), the first year of the COVID pandemic, was not decreased compared to previous years (103 TAB in 2019 and 140 in 2018).

**TABLE 1:** Temporal artery biopsy (TAB) study demographics.

Comparison	All	TAB positive	TAB negative	*P*-value
*n*	825	172 (21%)	653 (79%)	
Female: *n* (%)	549 (66%)	109 (63%)	440 (67%)	0.32^1^
Age: Median (IQR)	72 (65, 79)	76 (71, 81)	71 (63, 79)	<0.001^2^
Biopsy length (mm): Median (IQR)	14 (9, 18)	15 (10.5, 20)	13 (9, 18)	<0.001^2^

^1^Pearson’s chi-square.

^2^Wilcoxon rank sum.

**FIGURE 1:**
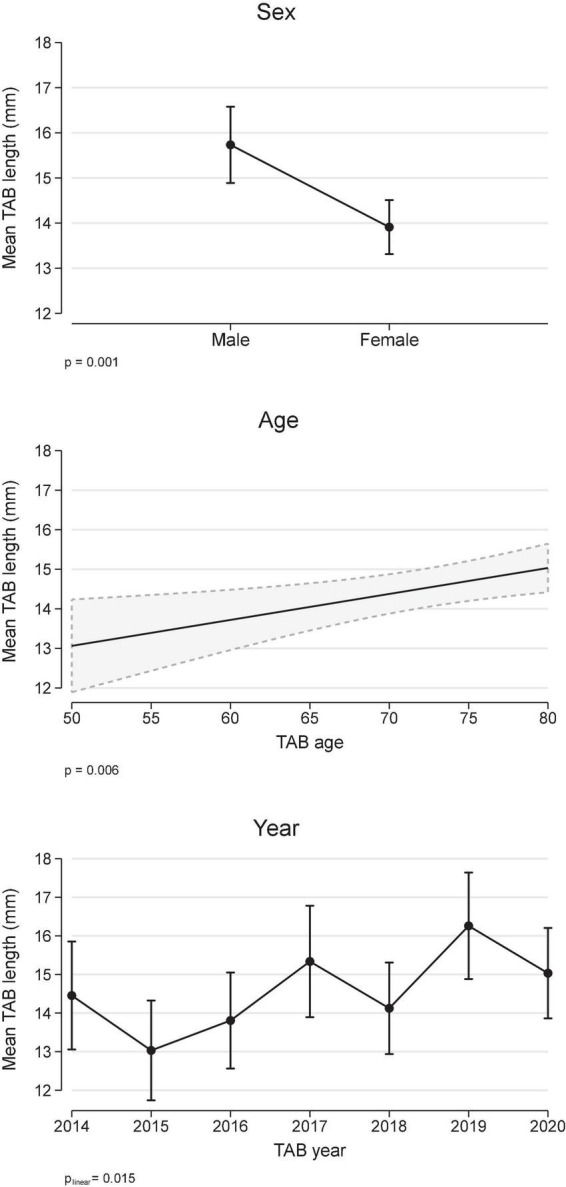
Temporal artery biopsy (TAB) length and covariates age, sex, and calendar year (multivariable linear regression). Results are expressed as marginal, population-averaged, outcome means.

The overall median biopsy length was 14 mm (IQR 9, 18). Analysis of biopsy length (mm) using a multivariable linear regression model (Figure 1) demonstrated that biopsy length was shorter in females compared to males (difference: −1.8 mm, 95% CI −2,9, −0.8), increased with older age (0.7 mm/decade, 95% CI 0.2, 1.1), and although variable, there was a smoothed linear trend toward increased length with increasing calendar year (*p* = 0.015). To put this in context, the difference in TAB length for 2017 onward compared to pre 2017 was 1.4 mm (95% CI 0.37, 2.35), after adjustment for age and sex.

**FIGURE 2:**
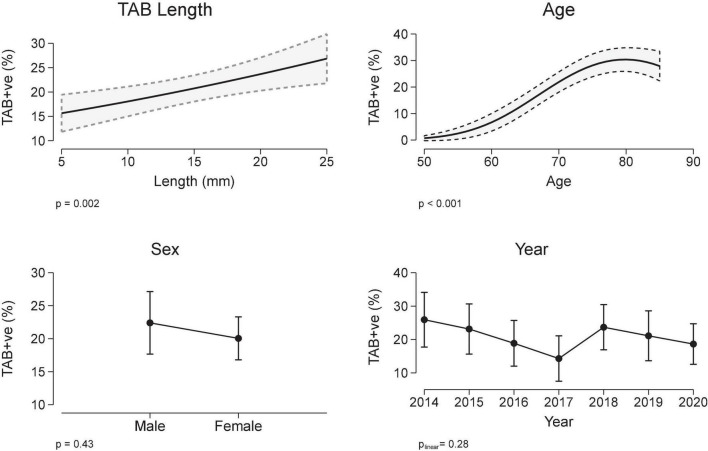
Temporal artery biopsy (TAB) positivity and covariates TAB length, sex, age, and year (multivariable logistic regression). Results are expressed as marginal, population-averaged, outcome means.

The relationship between a positive TAB and covariates sex, TAB age, TAB year and TAB length were determined using a multivariable logistic regression model, with the best model including a quadratic term for age. Patients with a positive TAB were more likely to be older (median age 76 vs. 71 years, Table 1 and Figure 2), but there was no association with either sex or TAB year (Figure 2). Importantly TAB length (mm) was associated with a positive TAB result in a linear manner. When adjusted for age, sex, and calendar year, the odds ratio for the association between a positive TAB result and TAB length was 1.04/mm, 95% CI 1.01, 1.06, *p* = 0.002, which equates in an increase in the marginal probability/proportion of a positive TAB result of 0.6%/mm (95% CI 0.2, 0.9, Figure 2).

Subsequent models compared the effect of dichotomizing TAB length at cut-points 5, 10, 15, and 20 mm on a TAB positive result (Table 2). While the differences were relatively small, the model with TAB length dichotomized at 15 mm was the best model with both the smallest AIC and largest AUC-ROC, however, less than half of the biopsies (383, 46%) in this study met this criteria. The diagnostic yield (positive predictive value) for TAB length ≥ 15 mm was 24.5% (95% CI20.4, 28.7), which, if all TAB had achieved this length, equates to an average of 30 additional individuals with a diagnosis of biopsy-proven GCA.

**TABLE 2:** The relationship between TAB length, dichotomised at 5, 10, 15, and 20 mm, and a positive TAB result.

	TAB length (mm)			
Descriptor	≥ 5 (vs. < 5)	≥ 10 (vs. < 10)	≥ 15 (vs. < 15)	≥ 20 (vs. < 20)
*n* (%)	798 (97%)	617 (75%)	383 (46%)	192 (23%)
OR (95% CI)	1.85 (0.53, 6.50)	1.66 (1.07, 2.59)	1.57 (1.10, 2.24)	1.52 (1.02, 2.25)
PPV (95% CI)	21.0% (18.3, 23.8)	22.6% (19.5, 25.8)	24.5% (20.4, 28.7)	26.0% (20.1, 31.9)
AUC-ROC (95% CI)	0.682 (0.640, 0.724)	0.689 (0.648, 0.731)	0.692 (0.649, 0.734)	0.690 (0.648, 0.732)
AIC	801.67	797.40	796.59	798.49

OR, odds ratios; PPV, positive predictive values; AUC-ROC, the area under the receiver operating curve; AIC, Akaike information criterion; were estimated from logistic regression models, adjusted for covariates sex, age, and calendar year.

## Discussion

A suspected diagnosis of GCA may be considered a medical emergency as early diagnosis with treatment intervention can prevent serious complications such as blindness and stroke. A GCA diagnosis is supported by a positive TAB, and increasingly, medical imaging technologies (2), which otherwise can be difficult or delayed because there are no laboratory findings specific for GCA and no particular signs or symptoms specific for the diagnosis. Exclusion of a GCA diagnosis is also important to prevent unnecessary exposure to the adverse effects associated with long-term corticosteroids.

With a specificity of 100%, a positive TAB remains an important tool for the diagnosis of GCA, yet it has a sensitivity of only 77% (5). Inadequate biopsy length has been identified in many, but not all, studies as a key factor in determining the diagnostic yield (sensitivity) of TAB for GCA diagnosis, attributable to “skip lesions” in the artery. Yet there is also a lack of consensus regarding the optimal TAB length. Reasons for these discrepancies may include the lack of standardization of biopsy harvesting, processing techniques and reporting, underlying differences in the TAB diagnostic yield due to differences in TAB referral (17), as well as a variable number of patients already on corticosteroid treatment at the time of biopsy.

In this study we have confirmed that there is a linear relationship between pre-fixation TAB length and the proportion of positive TAB results. Yet there are also practical constraints on the routinely achievable TAB lengths (particularly in females), and the recommended TAB length must balance the risk of biopsy with the probability of a positive result. Importantly, we identified that a TAB length of at least 15 mm was optimum for the predictive performance of our model, a result which is supported by two prior studies (18, 19). A high proportion of TAB lengths shorter than 15 mm may be characteristic of many studies, given that an overall mean length of 14.1 mm was estimated from a meta-analysis of 49 studies (4), but an increase to a minimum TAB length of 15 mm is realistic and achievable. To assess the potential impact of this, we estimated that a TAB length of at least 15 mm would have led to a positive TAB in an additional 30 patients who otherwise had a missed diagnosis or reduced treatment options, such as access to Tocilizumab which is reserved for biopsy positive cases in Australia.

Improved awareness of the importance of TAB length may not only decrease diagnostic and treatment delay, but may also obviate the need for a contralateral biopsy when there is a negative result, except when there is high index of clinical suspicion (15, 25). There was some evidence of such an improved awareness in our study, as we observed a small increase in biopsy length over the study duration, which has also been reported by some other studies over a longer time-frame (20, 26). We also observed, perhaps surprisingly, that TAB lengths increased with older age which could be consistent with an awareness of the importance of TAB in these patients who have a higher probability of GCA. In contrast, TAB length was shorter in females who also have a higher probability of GCA. This suggests that there may also be some anatomical or aesthetic constraints on TAB length. There is variation in site of TAB. Some surgeons preferring the common superficial temporal artery anterior to the tragus of the ear in favor of the frontal branch of the temporal artery. The difference in yield between these sites was not compared but might be useful to determine in future studies.

The strengths of this study are that it is a large study with all TAB processing and testing performed at a single laboratory which handles approximately 75% of TAB in South Australia. Therefore, it is a representative sample of suspected GCA cases in South Australia, without additional variability in sample processing and reporting. Some limitations include that there was no follow-up on the final clinical diagnosis, and therefore the effect of TAB length on the sensitivity/specificity of TAB for GCA diagnosis could not be evaluated. Further, there was no information on corticosteroid treatment at the time of biopsy, which may have also modified the relationship between TAB length and a positive TAB.

In conclusion, TAB remains a mainstay for a diagnosis of GCA, and TAB length is an important determinant of a positive result. We recommend a minimum pre-fixation length of 15 mm to obtain the maximum diagnostic yield for this procedure, yet approximately half of the biopsies in our study, and likely most published studies, did not meet this criterion. While there are some indications that awareness of the importance of TAB length is increasing amongst vascular or ophthalmologic surgeons, further emphasis is required. Standardization of TAB harvesting, processing techniques and reporting (including length) may also contribute to optimal diagnosis of biopsy-proven GCA.

## Data availability statement

Data is not available for privacy reasons. Requests to access the datasets should be directed to CH, catherine.hill@sa.gov.au.

## Ethics statement

The studies involving human participants were reviewed and approved by the Central Adelaide Local Health Network Human Research Ethics Committee. Written informed consent for participation was not required for this study in accordance with the national legislation and the institutional requirements.

## Author contributions

CR, CH, SLy, JN, and SLe: contribution to study conception and design. CR, JN, KD, SLe, TD, and CH: contribution to data acquisition. CR, JN, SLy, JT, RB, SLe, and CH: contribution to data analysis and interpretation. CR, JN, KD, SLy, JT, RB, TD, SLe, and CH: drafting the manuscript and critical revision and final approval of manuscript. All authors contributed to the article and approved the submitted version.
